# CRISPR/Cas9 Editing for Gaucher Disease Modelling

**DOI:** 10.3390/ijms21093268

**Published:** 2020-05-05

**Authors:** Eleonora Pavan, Maximiliano Ormazabal, Paolo Peruzzo, Emilio Vaena, Paula Rozenfeld, Andrea Dardis

**Affiliations:** 1 Regional Coordinator Centre for Rare Diseases, Academic Hospital of Udine, 33100 Udine, Italy; pavan.eleonora@gmail.com (E.P.); maxi.ormazabal@gmail.com (M.O.); paolo.peruzzo@asufc.sanita.fvg.it (P.P.); 2 Departamento de Ciencias Biológicas, IIFP, Universidad Nacional de La Plata, CONICET, Facultad de Ciencias Exactas Bv120 Nro.1489, La Plata 1900, Argentina; emiliovaena@gmail.com (E.V.); paularozenfeld@gmail.com (P.R.)

**Keywords:** Gaucher disease, cellular model, acid β-glucosidase, CRISPR/Cas9, unfolded protein response, neuroinflammation, α-synuclein, high-throughput drug screenings

## Abstract

Gaucher disease (GD) is an autosomal recessive lysosomal storage disorder caused by mutations in the acid β-glucosidase gene (*GBA1*). Besides causing GD, *GBA1* mutations constitute the main genetic risk factor for developing Parkinson’s disease. The molecular basis of neurological manifestations in GD remain elusive. However, neuroinflammation has been proposed as a key player in this process. We exploited CRISPR/Cas9 technology to edit *GBA1* in the human monocytic THP-1 cell line to develop an isogenic GD model of monocytes and in glioblastoma U87 cell lines to generate an isogenic GD model of glial cells. Both edited (*GBA1* mutant) cell lines presented low levels of mutant acid β-glucosidase expression, less than 1% of residual activity and massive accumulation of substrate. Moreover, U87 *GBA1* mutant cells showed that the mutant enzyme was retained in the ER and subjected to proteasomal degradation, triggering unfolded protein response (UPR). U87 *GBA1* mutant cells displayed an increased production of interleukin-1β, both with and without inflammosome activation, α-syn accumulation and a higher rate of cell death in comparison with wild-type cells. In conclusion, we developed reliable, isogenic, and easy-to-handle cellular models of GD obtained from commercially accessible cells to be employed in GD pathophysiology studies and high-throughput drug screenings.

## 1. Introduction

Gaucher disease (GD) is an autosomal recessive lysosomal storage disorder caused by mutations in the acid β-glucosidase gene (*GBA1*), resulting in the deficient activity of acid β-glucosidase (GCase), and the subsequent progressive accumulation of glucosylceramide (GlcCer) and glucosylsphingosine (LysoGL1) within the lysosomes, mainly in cells of the reticuloendothelial lineage [1,2]. 

In particular, accumulation of GlcCer in macrophages induces their transformation into engorged cells known as Gaucher cells, which infiltrate bone marrow, spleen and liver, and are considered key players in the development of the disease symptoms [3,4]. Indeed, the most prevalent manifestations of GD include hepatosplenomegaly, thrombocytopenia, anemia, coagulation abnormalities, and bone disease. In addition, some patients also present neurological manifestations [5,6].

The presence and severity/rate of progression of neurological involvement are discriminating factors for GD classification into three different clinical phenotypes, although the clinical picture presents as a phenotypic continuum. Type-1 GD, the most frequent phenotype, is the non-neuronopathic form of GD, whereas type-2 GD and type-3 GD are collectively referred to as neuronopathic GD (nGD), representing the acute and chronic neuronopathic phenotypes, respectively [7].

While hematological, visceral and skeletal manifestations of GD can be mainly explained by the infiltration of Gaucher cells, much less is known about the molecular mechanisms involved in neurodegeneration and/or neuronal cell death [8]. Studies performed on autopsy materials and on a mouse model of GD highlighted a specific pattern of neuron loss spatially and temporally correlated with neuroinflammation, i.e., microglial activation and astrocytosis [9,10]. Based on these findings, it has been hypothesized that GlcCer storage could trigger the activation of microglia and initiate a neuroinflammatory cascade involving elevation of cytokines and neurotoxic agents, which results in neuronal cell death [8]. In fact, inflammation seems to play an important role in the pathogenesis of GD, as significant changes in the levels of many inflammatory mediators, including interleukin-1β (IL-1β), are present not only in the serum of GD patients, i.e., released by macrophages [11,12] but also in the brain of GD mice [13,14]. 

Besides causing GD, *GBA1* mutations constitute the main genetic risk factor for developing Parkinson’s disease (PD), a neurodegenerative disorder characterized by α-synuclein (α-syn) accumulation and aggregation. Indeed, a strong association between GD and PD has been demonstrated, even though the molecular bases of this association remain elusive [15,16].

Research on the association between GD and PD has long been focusing on neurons, but a possible role of astrocytes has emerged [17]. Astrocytes, the most abundant glial cells, are active players in the viability and function of the central nervous system, taking part in the formation and maturation of synapses, receptor trafficking, control of the homeostasis of ions and energy metabolites and clearance of neurotransmitters, and their role in the onset and progression of many neurodegenerative diseases, including Alzheimer’s Disease, Huntington’s Disease, Amyotrophic Lateral Sclerosis and PD has been demonstrated [17,18,19]. Recently, the role of astrocytes also in the progression of nGD has been demonstrated by Aflaki et al. [20]. 

During the last decade, researchers have focused on the development of effective cellular models of GD which might reproduce the disease hallmarks genotypically and phenotypically, in order to pursue a better understanding of the pathophysiology and to develop novel therapeutic approaches [21,22]. 

The development of genome editing technologies, and in particular the CRISPR/Cas9 platform, has provided researchers with a versatile tool that can be exploited for the generation of cellular models of diseases by introducing site-specific mutations within the gene of interest [23,24]. The application of this technology offers the possibility to generate isogenic cells, generating models in which the only genetic difference between cell lines is the disease-causative mutation, avoiding the possibility of detecting non-disease-related phenotypes arising from differences in the genetic background of affected and control cells. In addition, the possible use of easily findable commercial cell lines allows the generation of disease models of different cellular types relevant to disease pathology.

To date, two in vitro models of GD have been generated exploiting CRISPR/Cas9 editing technology: Drews et al. [25] performed *GBA1* knock-out in HEK 293T cells and adenocarcinomic human alveolar basal epithelial A549 cells in order to study the role of GCase in influenza virus entry and infection. However, no models of disease have been developed employing cell lines relevant to study GD pathology. 

In this study, we exploited CRISPR/Cas9 technology for the generation of relevant cellular models reproducing GD hallmarks. Indeed, here we present two GD cellular models obtained by editing *GBA1*. After developing an editing workflow using Human Embryonic Kidney cells 293 (HEK), we edited a human monocytic cell line deriving from an acute monocytic leukemia patient (THP-1) and the glioblastoma U87 cell line (U87) to be used as a model of GD monocytes and glial cells, respectively.

## 2. Results

### 2.1. Monocytic GBA1 Mutant Cells: A Promising Cellular Model of GD

Gaucher cells, the lipid-laden storage macrophages, are the pathologic hallmark of GD. Therefore, we decided to perform *GBA1* editing in a human monocytic cell line deriving from an acute monocytic leukemia patient (THP-1). THP-1 can be easily differentiated into macrophage-like cells, which recapitulate several aspects of native monocyte-derived macrophages [26,27]. 

**Figure 1:**
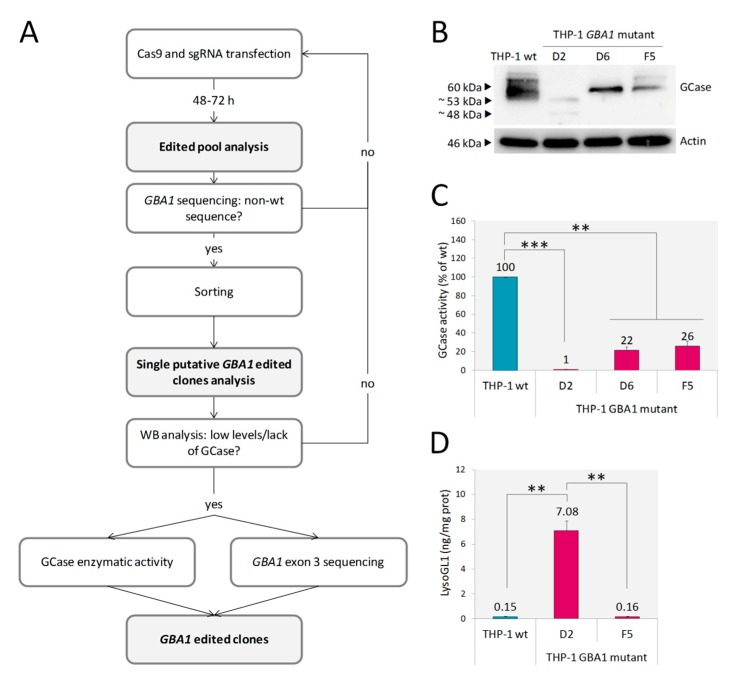
Development and characterization of THP-1 *GBA1* mutant cells. Schematic representation of *GBA1* editing workflow (**A**). WB analysis of THP-1 *GBA1* mutant D2, D6 and F5 clones showed lower levels of GCase expression in comparison to THP-1 wt (**B**). GCase enzymatic activity was significantly reduced in all three clones (**C**). A massive LysoGL1 accumulation was detected in THP-1 *GBA1* mutant clone D2 (**D**). Results are expressed as mean ± SD of three independent experiments. ** *p* < 0.001, *** *p* < 0.0001.

After applying the editing workflow (Figure 1A), three out of the 38 THP-1 clones screened by western blot (WB) (THP-1 *GBA1* mutant D2, D6 and F5) showed low expression of GCase (Figure 1B). Furthermore, in the case of clone D2, two proteins of a lower molecular weight (MW) in comparison with GCase wt were detected, suggesting that *GBA1* editing led to the generation of truncated proteins. Only clone D2 showed an almost absent enzymatic activity (residual activity = 1% of wild type), whereas GCase activity of D6 and F5 clones was 22% and 26%, respectively (Figure 1C). Then, we analyzed whether the reduction in GCase activity resulted in the accumulation of LysoGL1, as accumulated GlcCer in GD cells is converted by lysosomal acid ceramidase into its deacylated lysolipid, LysoGL1, which has been identified as an excellent biomarker of GD [28,29]. LysoGL1 was measured in clones D2 and F5 (Figure 1D). As expected, the 26% of residual activity retained by F5 was enough to prevent substrate accumulation in this clone, whereas, only the extremely low residual enzymatic activity of clone D2 led to a massive substrate accumulation. Therefore, clone D2 was selected as a model of GD. Sequencing analysis of the genomic DNA of the D2 clone showed the absence of wt sequence and two large deletions involving *GBA1* exon 3 (Figure S1). These alterations likely result in the in-frame exclusion of exon 3 and exons 3/4 from the mature mRNA, respectively, consistent with the expression of the two proteins of ~53 and 48 kDa respectively, observed by WB analysis (Figure 1B). 

### 2.2. U87 GBA1 Mutant Cells: A Useful Tool to Study Neuroinflammation in GD

Together with a few additional glioma cell lines, glioblastoma U87 cell line (U87) is probably the most commonly used cell line in research on human gliomas [30]. It can give rise to epithelial-glial cells, i.e., astrocytes, oligodendrocytes or ependymal cells [31,32]. In order to investigate the contribution of glial cells, beyond the macrophage-derived glia (microglia), to GD pathology, we generated a model of GD by editing *GBA1* in the U87 cell line to investigate their possible role in GD pathogenesis. 

**Figure 2:**
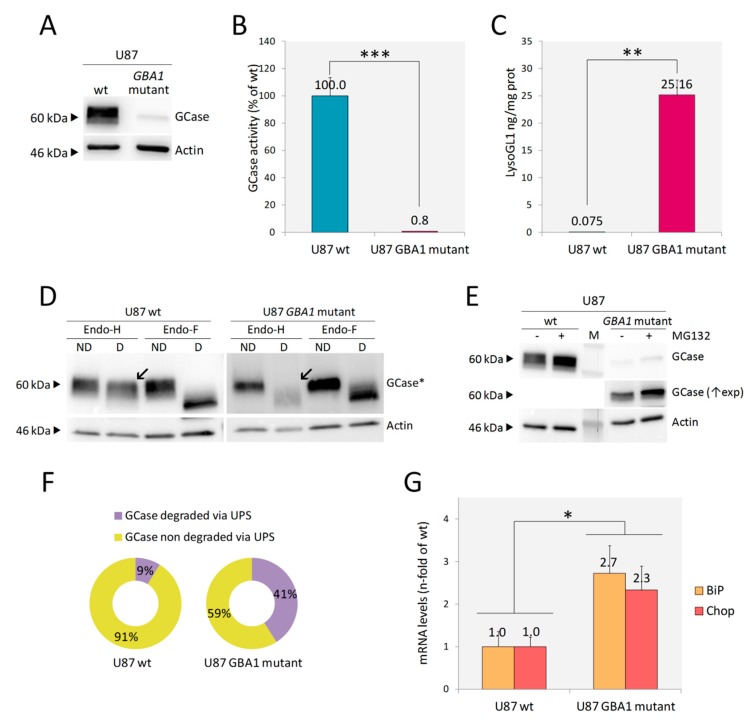
Development and characterization of U87 *GBA1* mutant cells. WB analysis of U87 *GBA1* mutant cells showed lower levels of GCase expression in comparison to U87 wt (**A**). A significant reduction of GCase enzymatic activity (**B**), as well as LysoGL1 accumulation (**C**), were detected. When digested by Endo-H, U87 *GBA1* mutant showed lack of mature GCase (indicated by arrows in **D**); the Endo-F cleavage pattern confirmed that difference in protein migration on SDS-PAGE was only due to protein glycosylation [*GCase detection was performed at differential exposure time between U87 wt and U87 *GBA1* mutant] (**D**). A significant increase of GCase protein abundance was detected in cells treated with the proteasome inhibitor MG132 by WB (**E**). The quantification of GCase signals, normalized to actin, in the presence and absence of MG132 showed that 32% of the mutant protein was subjected to proteasomal degradation (**F**). mRNA levels of ER stress markers BiP and Chop were significantly increased in U87 *GBA1* mutant cells in comparison to U87 wt (**G**). Results are expressed as mean ± SD of three independent experiments. * *p* < 0.05, ** *p* < 0.001, *** *p* < 0.0001. Abbreviations: M = marker, ND = non digested, D = digested, (↑exp) = longer exposure.

Following the canonical editing workflow, several clones were screened by WB. Most of them did not express any GCase protein. Enzymatic activity assessed in two of them was undetectable (data not shown). In addition, as reported in Figure 2A,B, one clone presented a very low expression of GCase and retained <1% of GCase enzymatic activity. This clone (U87 *GBA1* mutant) was selected for further characterization since it would offer the possibility to study, in addition to the phenotypic changes associated with substrate accumulation, the possible effects mediated by the expression of a mutant protein, such as Endoplasmic reticulum associated degradation (ERAD) and unfolded protein response (UPR). Sequencing analysis of the genomic DNA showed a large deletion involving the whole *GBA1* exon 2 and the first part of an exon in compound heterozygosis with an in frame six base pair deletion in *GBA1* exon 3 (Figure S2). The large deletion would result in the exclusion of the entire exons 2 and 3 with the consequent shifting in the reading frame and the generation of a premature stop codon within exon 4. The transcript or the putative 12 amino acid peptide generated by this allele would be rapidly degraded. Instead, the allele carrying the in frame six base pair deletion gives rise to a protein lacking in Thr82 and Arg83, which seems to have the same MW of GCase wt (~60 kDa), as shown by WB analysis (Figure 2A). Any off-target effect of the guide was excluded by sequencing off-target regions identified by the modified version of the algorithm presented by Zhang et al. [33] (Table S1). 

As expected, a massive accumulation of LysoGL1 was found in U87 *GBA1* mutant cells (Figure 2C). 

#### 2.2.1. Endoplasmic Reticulum Associated Degradation (ERAD) and ER Stress

Many *GBA1* mutations identified in GD patients cause the synthesis of misfolded proteins that are retained in the endoplasmic reticulum (ER) and subjected to ER-associated degradation (ERAD), which involves their translocation to the cytosol and the elimination by the ubiquitin-proteasomal pathway [34]. Therefore, we investigated whether the mutant GCase synthesized by the U87 *GBA1* mutant clone was subjected to ERAD. To this aim, we first analyzed the GCase glycosylation status by treating protein lysates with Endoglycase H (Endo-H) and Endoglycase F (Endo-F). As the first enzyme cleaves asparagine-linked mannose rich oligosaccharides, but not highly processed complex oligosaccharides from glycoproteins, it can distinguish between glycoproteins that have not reached the mid-Golgi (sensitive to Endo-H digestion), and processed mature glycoproteins (non-sensitive to Endo-H digestion). Endo-F removes all asparagine-linked glycans, and so, its cleavage pattern was used as a control to confirm that the changes in protein migration on SDS-PAGE were only due to protein glycosylation [34].

The Endo-H cleavage pattern confirmed that the whole amount of GCase expressed by the mutant clone was retained in the ER: Endo-H treated lysate showed the same cleavage pattern as Endo-F treated lysate, and no differences in migration on SDS-PAGE were recorded, according to Endo-F cleavage pattern (Figure 2D). Then, we analyzed whether the ER retained GCase was subjected to proteasomal degradation by assessing the protein levels in the *GBA1* mutant cells treated with MG132, a proteasome inhibitor. As shown in Figure 2E,F, proteasome inhibition led to a significant increase (32%) of GCase protein abundance, meaning that the p.Thr82_Arg83del mutant GCase undergoes extensive proteasomal degradation. Taken together, these data suggest that in U87 *GBA1* mutant cells, the mutant GCase is unfolded, retained in ER and subjected to ERAD.

Then, we wanted to investigate whether the presence of the unfolded protein in the ER led to ER stress triggering unfolded protein response (UPR). To this aim, we assessed the mRNA levels of two markers: BiP, a major ER chaperon induced by ER stress, and Chop, a UPR target gene involved in ER stress-mediated apoptosis. Both transcripts were significantly increased in U87 *GBA1* mutant cells in comparison to U87 wt, confirming UPR activation in these cells (Figure 2G).

#### 2.2.2. Inflammatory Markers

**Figure 3:**
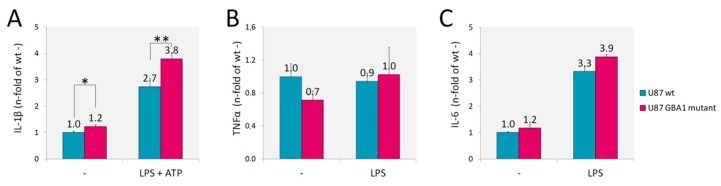
Inflammatory markers. Cytokines levels evaluated by capture ELISA in the supernatant of U87 *GBA1* mutant cells showed an increased production of IL-1β without (-) or with (LPS + ATP) inflammosome activation (**A**). No changes in TNFα (**B**) nor IL-6 (**C**) were detected, with or without LPS stimuli. Results are expressed as mean ± SD of three independent experiments. * *p* < 0.05, ** *p* < 0.001.

Since inflammation is a key player in the pathogenesis of GD, we assessed U87 *GBA1* mutant cells for inflammatory cytokines whose increase has been observed in patients serum and in several GD models [12,13,14,35,36]. In addition, inflammation and neuroinflammation have been recently demonstrated also in a fly model of GD [37]. We found increased production of interleukin-1β (IL-1β) (Figure 3A), but no production of tumor necrosis factor α (TNFα) nor interleukin-6 (IL-6) (Figure 3B,C) by U87 *GBA1* mutant cells. IL-1β secretion by this GD model implies a constitutive activation of inflammasome. Indeed, when we activated inflammasome by adding LPS and ATP, a higher increase of IL-1β production was detected.

#### 2.2.3. Accumulation of α-Synuclein and Cell Death

**Figure 4:**
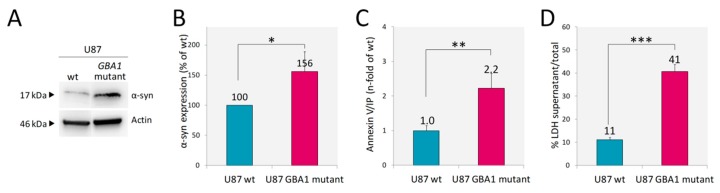
Accumulation of α-synuclein and cell death. The higher α-syn abundance shown by the U87 *GBA1* mutant by WB analysis (**A**). Quantification of α-syn signals normalized to actin (**B**) showed that mutant cells displayed a 56% increase in α-syn content. This increment was accompanied by an increased percentage of apoptotic cells as assessed by Annexin V positive and propidium iodide (IP) negative (**C**) and LDH activity release (**D**). Results are expressed as mean ± SD of three independent experiments. * *p* < 0.05, ** *p* < 0.001, *** *p* < 0.0001.

Recent data suggest that astrocytes may contribute, at least in part, to the mechanism linking GD and PD [20]. Therefore, we analyzed α-syn expression. U87 cells even under normal conditions express detectable levels of monomeric α-syn and the presence of mutant inactive GCase resulted in a significant increase of α-syn abundance, as reported in Figure 4A,B. A recent report revealed that the presence of mutant GCase both in SHSY5Y cells and in Drosophila dopaminergic cells leads to accumulation and aggregation of α-syn and to faster death of these cells [38]. Given the finding of increased levels of α-syn in our U87 model, we aimed to analyze if it was accompanied by increased cell death. In the culture of U87 *GBA1* mutant cells, we found an increased percentage of apoptotic cells, as assessed by Annexin V/IP in comparison to U87 wt (Figure 4C). Moreover, cell death was more prominent in the mutant cells as shown by the release into the cell culture medium of the cytosolic enzyme lactate dehydrogenase (LDH) associated with damage of the plasma membrane (Figure 4D).

## 3. Discussion

CRISPR/Cas9 editing technology is a powerful and versatile tool for genetic manipulation, due to its high specificity and efficiency, easiness of application and low costs. For these reasons, it has been already employed to generate cellular models of many sphingolipidoses, as review by Santos R and Amaral O [23]. Here, we have outlined a CRISPR/Cas9 *GBA1* editing workflow based on the use of commercial editing tools without the need for plasmid-cloning steps. The protocol comes as technically easy to perform and efficiently applicable to both adhesion- and suspension-growing cells. Indeed, the described workflow has successfully been used to edit *GBA1* in U87 and THP-1 cells.

In GD research, RNA interference (RNAi) technologies or pharmacological inhibition with Conduritol β Epoxide have been commonly chosen to recreate disease hallmarks. However, their effect is transient and partial. More recently, protocols to efficiently obtain GD macrophages, neurons and astrocytes derived from induced pluripotent stem cells (iPSC) have been developed [20,39,40,41]. However, these protocols are usually time-consuming and technically challenging and frequently not suitable for large scale production of cells for drug screenings. In addition, the main drawback of using cells obtained from iPSC derived from patients and healthy controls for comparative studies is the possibility to detect differences due to the different genetic background of the cells [42,43]. 

On the contrary, gene disruption mediated by CRISPR/Cas9 technology is permanent and complete, and thus, repetitive administration is not required [44]. In addition, the application of editing technologies to commercial cell lines offers the opportunity to easily generate isogenic cells, avoiding confounding results due to different genetic backgrounds. For these reasons, we edited *GBA1* in two cell types that are relevant for disease pathology: U87 (as a model of glial cells) and THP-1 (as a model of monocytes). Differently from GCase-null HEK and A549 cells developed by Drews et al. [25], both THP-1 and U87 *GBA1* mutant clones do express mutant but non-functional forms of GCase.

We validated the reliability of our models in terms of GCase expression and activity, as well as LysoGL1 accumulation. Indeed, both *GBA1* mutants generated cell lines presented lower levels of mutant GCase expression, less than 1% of residual GCase activity and massive accumulation of LysoGL1. Therefore, they recapitulate the main hallmarks of GD.

Once established the reliability of THP-1 and U87 *GBA1* mutant cells, the range of their possible applications is extremely wide. 

THP-1 *GBA1* mutant cells represent an excellent model to further explore GD pathophysiology. Indeed, since they can be differentiated into macrophage-like cells miming native monocyte-derived macrophages in several aspects, they may compensate for the lack of appropriate cell-based models exhibiting glycolipid storage analogous to that seen in patient macrophages, with all the advantages of using an isogenic continuous cell line. Indeed, they represent a good model to study GD pathophysiology as an alternative to cells much more difficult to obtain and to expand in vitro, such as patients derived monocytes, monocytes-differentiated macrophages or iPSC-derived macrophages.

The role of glial cells such as astrocytes, in the pathogenesis of GD, with a particular focus on neuroinflammation, seems to be a promising but under investigated aspect of GD [20]. Therefore, we decided to perform a deeper characterization of the newly generated U87 *GBA1* mutant cells. 

The contribution of protein misfolding and ER retention of mutant GCase in the pathogenesis of GD has been already extensively described. The presence of misfolded GCase retained in the ER has two main consequences: on one hand, it is usually retrotranslocated from the ER to the cytosol, where it undergoes degradation by the ubiquitin–proteasome system (UPS); on the other hand, it is thought to cause ER stress eventually triggering the UPR. Indeed, both UPS degradation and UPR existence have been reported in skin fibroblasts from GD patients [45,46,47,48,49]. However, UPR has not been explored in human GD glial cells. In this study, we have shown that besides being retained in the ER and subjected to proteasomal degradation, mutant GCase triggers UPR in the U87 *GBA1* mutant model.

In addition, an increase in interleukin-1β (IL-1β) release from these cells was observed. This finding is consistent with the significant increase of many inflammatory mediators in the serum of GD patients and in the brain of a neuronal model of GD mice [11,12,13,14,50]. 

All these features, namely substrate accumulation, mutated GCase and neuroinflammation, have been linked to α-syn accumulation [51,52,53,54,55,56]. Indeed, U87 *GBA1* mutant cells display α-syn accumulation as well. Therefore, these cells seem to be a good model to investigate the relative contribution of each of these mechanisms to the relationship between GCase and α-syn. 

Finally, U87 *GBA1* mutant cells displayed increased levels of apoptosis and cell death, which might be triggered by UPR activation, neuroinflammation and α-syn accumulation, all processes that are altered in *GBA1* mutant cells. Thus, this model represents an excellent tool to also explore the role of these cellular processes in GD pathology.

Besides presenting the main hallmarks of GD, the U87 *GBA1* mutant cells are easy-to-handle and suitable for large scale production of cells. Therefore, they are ideal to perform high throughput screening of drugs that target the plethora of molecular pathways found to be altered in GD.

**Figure 5:**
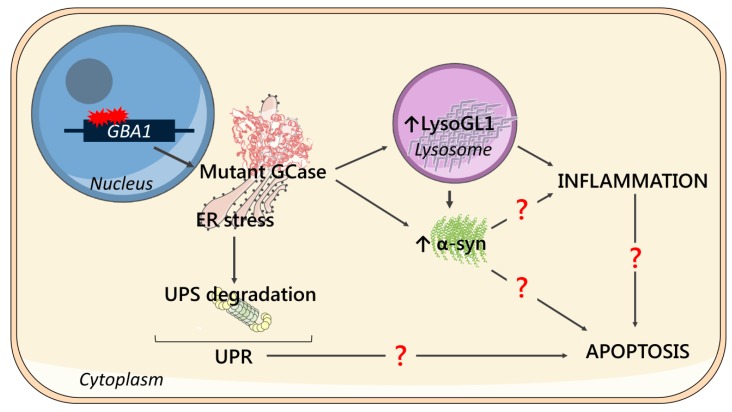
Schematic representation of the main cellular features and their possible correlation. Mutations in the *GBA1* gene cause the synthesis of a mutant form of GCase, which is retained in the ER causing ER stress and subjected to degradation via the ubiquitin-proteasome system (UPS degradation), triggering unfolded protein response (UPR). The low enzymatic activity of the mutant GCase leads to LysoGL1 accumulation in lysosomes, which has been associated with neuroinflammation. Mutated GCase and substrate storage might lead to α-syn accumulation, which may have a direct role in the development of inflammation. Moreover, α-syn accumulation, UPR and/or inflammation may be the key triggers of apoptotic processes, eventually causing cell death.

As a summary, a schematic representation of the main cellular features and their possible correlation identified in this work is shown in Figure 5. 

In conclusion, we obtained models of two cellular types relevant for GD pathology that recapitulate the main hallmarks of the disease, using CRISPR/Cas9 editing technology: THP-1 *GBA1* mutant monocytes and U87 *GBA1* mutant glial cells. These models will be useful tools to investigate GD pathophysiology and to identify novel potential treatments through high-throughput drug screenings.

## 4. Materials and Methods 

### 4.1. Cell Culture 

Human embryonic kidney cells 293 (HEK) were cultured and maintained in Dulbecco’s modified Eagle’s medium high glucose (EuroClone, Pero, Italy) containing 10% fetal bovine serum (Gibco-Thermo Fisher Scientific, Waltham, MA, USA), 1% glutamine (Gibco-Thermo Fisher Scientific, Waltham, MA, USA), and 1% penicillin/streptomycin (Gibco-Thermo Fisher Scientific, Waltham, MA, USA), in a humidified atmosphere containing 5% CO_2_ at 37 °C. 

Glioblastoma U87 cells (U87) were cultured and maintained in Dulbecco’s modified Eagle’s medium low glucose (EuroClone, Pero, Italy) containing 10% fetal bovine serum (Gibco-Thermo Fisher Scientific, Waltham, MA, USA), 1% glutamine (Gibco-Thermo Fisher Scientific, Waltham, MA, USA), and 1% penicillin/streptomycin (Gibco-Thermo Fisher Scientific, Waltham, MA, USA), in a humidified atmosphere containing 5% CO_2_ at 37 °C. 

Human monocytic cells deriving from an acute monocytic leukemia patient (THP-1) were cultured and maintained in RPMI 1640 medium (EuroClone, Pero, Italy) containing 10% fetal bovine serum (Gibco-Thermo Fisher Scientific, Waltham, MA, USA), 1% glutamine (Gibco-Thermo Fisher Scientific, Waltham, MA, USA), and 1% penicillin/streptomycin (Gibco-Thermo Fisher Scientific, Waltham, MA, USA), in a humidified atmosphere containing 5% CO_2_ at 37 °C.

### 4.2. MG132 Treatment

In order to investigate the relative amount of GCase degraded via the ubiquitin-proteasome system, cells were treated with 15 µM of the proteasomal inhibitor MG132 (Sigma-Aldrich, St. Louis, MO, USA) for 24 h; protein extracts from treated and not treated cells were then separated on a 4%–20% gradient mini-protean TGX pre-cast gel (BioRad, Hercules, CA, USA) and western blot analysis was performed using anti-GBA 2E2 antibody.

### 4.3. CRISPR/Cas9 GBA1 Editing

*GBA1* editing was performed using Invitrogen TrueGuide Synthetic guide RNA (A35510-CRISPR813153_SG) (sgRNA) and TrueCut™ Cas9 Protein v2, with Lipofectamine™ CRISPRMAX™ Cas9 transfection reagent (Invitrogen-Thermo Fisher Scientific, Waltham, MA, USA), according to the manufacturer’s protocols. Briefly, cells were seeded one day before transfection in 24-well plate (HEK: 80 × 10^3^ cell/well; THP-1: 50 × 10^3^ cell/well; U87: 80 × 10^3^ cell/well). Transfection was performed using 1250 ng of Cas9 protein, 7.5 pmols of sgRNA, 2.5 µL of Lipofectamine™ Cas9 plus reagent and 2 µL of Lipofectamine™ CRISPRMAX™ reagent in HEK and U87 cells, or 2000 ng of Cas9 protein, 12 pmols of sgRNA, 4 µL of Lipofectamine Cas9 plus reagent and 1.5 µL of Lipofectamine™ CRISPRMAX™ reagent in THP-1 cells. After 48–72 h the edited pool was sorted by single-cell sorting in flow cytometry (BD FACSAria III, BD Biosciences, San Jose, CA, USA).

With the purpose of setting up an editing protocol, we tested CRISPR/Cas9 technology on Human embryonic kidney cells 293 (HEK), which have been widely used in research because of their reliable growth and predisposition for transfection [57].

Briefly, after 48–72 h form sgRNA and Cas9 transfection, we sequenced *GBA1* exon 3 of the edited pool, and when we noticed non-wild type sequence, we proceeded to single cell sorting in flow cytometry. Then, we analyzed every single clone by Western blot (WB) in order to detect lack or low levels of GCase: if so, we fully characterized the clone by measuring GCase activity and sequencing *GBA1* exon 3 (Figure 1A).

### 4.4. Off-Target Analysis 

Off-target analysis of sgRNA was performed using a modified version of the algorithm presented by Zhang et al. [33]. Off-target site prediction was performed using the CRISPOR tool V4.2 (http://crispor.org) and the CCTop tool (http://crispr.bme.gatech.edu); the third tool (CRISPR design tool, http://crispr.mit.edu) presented by Zhang et al. was not used since the website was shut down. All exonic off-target sites predicted by any of the two tools were considered, whereas intronic off-target sites were taken into account when predicted by both tools. *GBA1* pseudogene (*GBAP1*) was excluded from the analysis as *GBA1-GBAP1* rearrangements were considered as advantageous to edit *GBA1*. This analysis led to the identification of five exonic and nine intronic regions, reported in Table S1. A section of ~400 bp flanking the sgRNA target site was PCR amplified using GoTaq (Promega, Madison, WI, USA) and F and R primer for each gene as reported in Table S2. Amplification was performed according to the following protocol: 95 °C 2 min; 35 cycles consisting of 95 °C 30 s, Ta °C 30 s, 72 °C 30 s; 72 °C 7 min. PCR products were purified using Illustra ExoProStar clean up (GE Healthcare Life Sciences, Chicago, IL, USA) according to the manufacturer’s protocols. BigDye (Applied Biosystem-Thermo Fisher Scientific, Waltham, MA, USA) and each F and R primer (Table S2) were used in the sequencing reaction (26 cycles consisting of 95 °C 10 s, 50 °C 15 s, 62 °C 2 min). Sequences were purified in EtOH 70% and loaded in 3500xL Genetic Analyzer (Applied Biosystems-Thermo Fisher Scientific, Waltham, MA, USA). Sequencing analysis was performed using Chromas software (version 2.6.6, Technelysium Pty Ltd., South Brisbane, QLD, Australia). 

### 4.5. DNA Extraction, GBA1 Exon 3 Amplification and Sequencing 

DNA was extracted from putative *GBA1* edited clones in order to perform sequence characterization using a DNeasy blood and tissue kit (Qiagen GmbH, Hilden, Germany) according to the manufacturer’s protocols. 

*GBA1* exon 3 and exon 3 flanking regions were PCR amplified using Platinum™ Taq DNA Polymerase high fidelity (Invitrogen-Thermo Fisher Scientific, Waltham, MA, USA) and primers 1F and 5R (Table S2) to selectively amplify the gene and not the homologous pseudogene. Amplification was performed according to the following protocol: 94 °C 2 min; 10 cycles consisting of 94 °C 10 s, 57 °C 30 s, 68 °C 4 min; 20 cycles consisting of 94 °C 10 s, 57 °C 30 s, 68 °C 4 min, adding 20 s at each cycle; 68 °C 7 min. PCR products were purified from gel (1% agarose in TBE) using QIAquick gel extraction kit (Qiagen GmbH, Hilden, Germany) and cloned using a TOPO-TA cloning kit (Thermo Fisher Scientific, Waltham, MA, USA), according to the manufacturer’s protocols. BigDye (Applied Biosystem-Thermo Fisher Scientific, Waltham, MA, USA) and primers 3F and 3R (Table S2) were used in sequencing reaction (26 cycles consisting of 95 °C 10 s, 50 °C 15 s, 62 °C 2 min). Sequences were purified in EtOH 70% and loaded in a 3500xL Genetic Analyzer (Applied Biosystems-Thermo Fisher Scientific, Waltham, MA, USA). Sequencing analysis was performed using Chromas software (version 2.6.6, Technelysium Pty Ltd., South Brisbane, QLD, Australia). *Accession numbers of RNA and protein sequences: NM_000157.3, NP_000148.2.*

### 4.6. Protein Extraction and Western Blot 

In order to evaluate expression levels of GCase and α-syn, cells were washed once with PBS and directly lysed in cell Lysis Buffer TNN (Tris-HCl 100 mM pH 8, NaCl 250 mM, NP40 0.5%), sonicated and incubated in ice for 10 min. After centrifugation (10 min at full speed at 4 °C), protein extracts were analyzed for protein content using the Bradford assay, using the BioRad protein assay (BioRad, Hercules, CA, USA), following manufacturer’s instructions.

Protein extracts were resolved in sodium dodecyl sulfate-polyacrylamide gel electrophoresis (SDS-PAGE). Equal lysate amounts per lane were loaded on a 4%–20% gradient mini-protean TGX pre-cast gel (BioRad, Hercules, CA, USA) in running buffer (running buffer 10 X: Tris 25 mM, Glycine 0.191 M, SDS 0.1% *w*/*v*). Fractionated proteins were transferred to nitrocellulose membrane (BioRad, Hercules, CA, USA) in transfer buffer (Tris 25 mM, Glycine 0.189 M, 40% MeOH), and after blocking with paraformaldehyde (PFA) 0.4% in phosphate-buffered saline (PBS, Euroclone, Pero, Italy) for 30 min., membranes were blocked in 5% blotting-grade blocker (BioRad, Hercules, CA, USA) in PBS-T (0.1% Tween 20 in PBS) for 1 h. Then, membranes were incubated overnight at 4 °C with the appropriate primary antibody 1:1000 (GBA 2E2 (WH0002629M1, Sigma-Aldrich, St. Louis, MO, USA), α-syn211 (sc-12767, Santa Cruz Biotechnology, Dallas, TX, USA), actin (A2066, Sigma-Aldrich, St. Louis, MO, USA)), then washed, incubated with the appropriate secondary antibody (Dako Agilent, Santa Clara, CA, USA) for 1 h at RT and developed with SuperSignal West Dura/Pico reagents (Thermo Fisher Scientific, Waltham, MA, USA). The total expression of the protein of interest was normalized to actin levels. Blots were quantified by using a Uvitec Cambridge Imaging system (UVITEC, Cambridge, UK).

### 4.7. Endo-H and Endo-F Digestion 

With the purpose of distinguishing between glycoproteins that have not reached the mid-Golgi and folded, processed, mature glycoproteins, cell lysates containing 30 µg of total protein were subjected to overnight incubation at 37 °C with 5 µL of Endo-H or 2.5 µL of Endo-F (Roche, Basel, Switzerland) upon denaturing the samples for 5 min. at 95 °C in Endo-H or Endo-F denaturing buffer (Endo-H Buffer: buffer sodium citrate 50 mM pH 5.2, PMSF 0.5 mM, SDS 0.1%, Triton X 100 0.5%, mercaptoethanol 0.1 M; Endo-F Buffer: Buffer sodium phosphate 100 mM pH 7.2, EDTA 10 mM, Triton X 100 0.1%, SDS 0.1%, mercaptoethanol 1%). Digested and undigested samples were then separated on a 4%–20% gradient mini-protean TGX pre-cast gel (BioRad, Hercules, CA, USA) and western blot analysis was performed using anti-GBA 2E2 antibody, as described above.

### 4.8. Enzymatic Activity

GCase enzymatic activity was measured using the fluorogenic substrate 4-methylumbelliferyl-β-d-glucopyranoside (Sigma-Aldrich, St. Louis, MO, USA); the total amount of protein in cell lysates was determined using the Bradford assay, using the BioRad protein assay (BioRad, Hercules, CA, USA), following manufacturer’s instructions.

Briefly, 10 µL containing 10 µg of protein was incubated with 10 μL of substrate 5 mM in acetate buffer 0.1 M pH 4.2 at 37 °C for 3 h. The reaction was stopped with carbonate buffer 0.5 M pH 10.7 and the fluorescent product was quantified using a fluorimeter (SPECTRAmax Gemini XPS, Molecular Devices, San Jose, CA, USA) at an excitation wavelength of 365 nm and emission of 495 nm. 

### 4.9. RNA Extraction, Reverse Transcription and Quantitative Real Time PCR 

Total RNA was isolated using the QIA Shredder and the RNeasy mini kit (Qiagen GmbH, Hilden, Germany). First strand cDNA synthesis was performed with 1 μg total RNA using random hexanucleotides and MMLV reverse transcriptase (Invitrogen-Thermo Fisher Scientific, Waltham, MA, USA). Primers were designed from available human sequences using the primer analysis software Primer3 (Table S2). Quantitative RT-PCR was performed using SYBR Green PCR master mix (Invitrogen-Thermo Fisher Scientific, Waltham, MA, USA) in iQ-Cycler equipment (BioRad, Hercules, CA, USA), following the manufacturer’s instructions. A human β-actin gene was used as an internal control. The comparative threshold (C_t_) method was used for data analysis expressed as 2^–Δ*C*t^.

### 4.10. LysoGL1 Measurement

LysoGL1 in cell pellets was measured using a published LC-MS/MS method previously described [58]. D5-glucosylsphingosine was used as an internal standard. LysoGL1 is separated from its isomer galactosylsphingosine (psychosine) using an Acquity UPLC BEH amide column (2.1 mm × 100 mm with 1.7 μm particle size). An Agilent 6490 triple quadrupole mass spectrometer coupled with an Agilent 1290 liquid chromatography system was used for the analysis.

### 4.11. Cytokine Measurement

Cytokine levels in the supernatant fraction were quantified by capture ELISA (BD Biosciences, San Jose, CA, USA) following the manufacturer instructions. U87 cells were grown in 24-well plates containing approximately 0.2 × 10^6^ cells/well at the time of experimentation. To stimulate the inflammasome to release IL-1β cytokine, cells were treated with LPS (Millipore, Darmstadt, Germany) and ATP (Sigma-Aldrich, St. Louis, MO, USA) at a final concentration of 100 ng/mL and 5 mM, respectively. For stimulation of TNF-α and IL-6 cytokine release, cells were treated with LPS only at the same concentration described above.

### 4.12. Annexin-V Staining

U87 cells were harvested by treatment with TrypLE solution (Gibco-BRL, Carlsbad, CA, USA) and washed with fresh medium. Cells were centrifuged and resuspended in 50 µL of binding buffer (HEPES 10 mM, NaCl 140 mM, CaCl_2_ 2.5 mM, pH = 7.40); then, 1 µL of Annexin V-APC (BD Pharminigen, San Diego, CA, USA) and 1 µL of propidium iodide (1 mg/mL) (Sigma-Aldrich, St. Louis, MO, USA) were added. After a 30-min incubation at room temperature, samples were analyzed by flow cytometry in a FACScalibur (Becton Dickinson, Franklin Lakes, NJ, USA).

### 4.13. Lactate Dehydrogenase (LDH) Activity

To evaluate U87 necrotic cells we used a lactate dehydrogenase (LDH) assay because of the release of LDH from cells into the culture medium upon the rupture of the plasma membrane. LDH was measured with a commercially available LDH assay kit (LDH Kit, Wiener Lab, Rosario, Argentina) according to the manufacturer´s instructions. LDH supernatant activity was expressed as a percentage, taking as 100% the LDH from lysed cells plus LDH in the supernatant.

### 4.14. Statistical Analysis

Statistical significance was determined by Student’s *t*-test; *p* < 0.05 was considered as statistically significant.

## Supplementary Materials

Supplementary materials can be found at https://www.mdpi.com/1422-0067/21/9/3268/s1.

## Abbreviations

Endo-F	Endoglycase F
Endo-H	Endoglycase H
ER	Endoplasmic reticulum
ERAD	ER associated degradation
GCase	acid β-glucosidase
GD	Gaucher disease
GlcCer	Glucosylceramide
HEK	Human Embryonic Kidney cells 293
IL-1β	Interleukin-1β
IL-6	Interleukin-6
iPSC	Induced pluripotent stem cells
LDH	Lactate dehydrogenase
LysoGL1	Glucosylsphingosine
MW	Molecular weight
nGD	Neuronopathic GD
PD	Parkinson’s disease
RNAi	RNA interference
THP-1	Human monocytic cell line deriving from an acute monocytic leukemia patient
TNFα	Tumor necrosis factor α
U87	Glioblastoma U87 cell line
UPR	Unfolded Protein Response
UPS	Ubiquitin-Proteasomal System
WB	Western Blot
α-syn	α-synuclein
