# Supplementary material for: CRISPR/Cas9 Editing for Gaucher Disease Modelling
Source: Int J Mol Sci. 2020 May 5;21(9):3268. doi: 10.3390/ijms21093268 (PMC7246564; doi:10.3390/ijms21093268)
Supplement: Supplementary file 1 [file ijms-21-03268-s001.pdf]

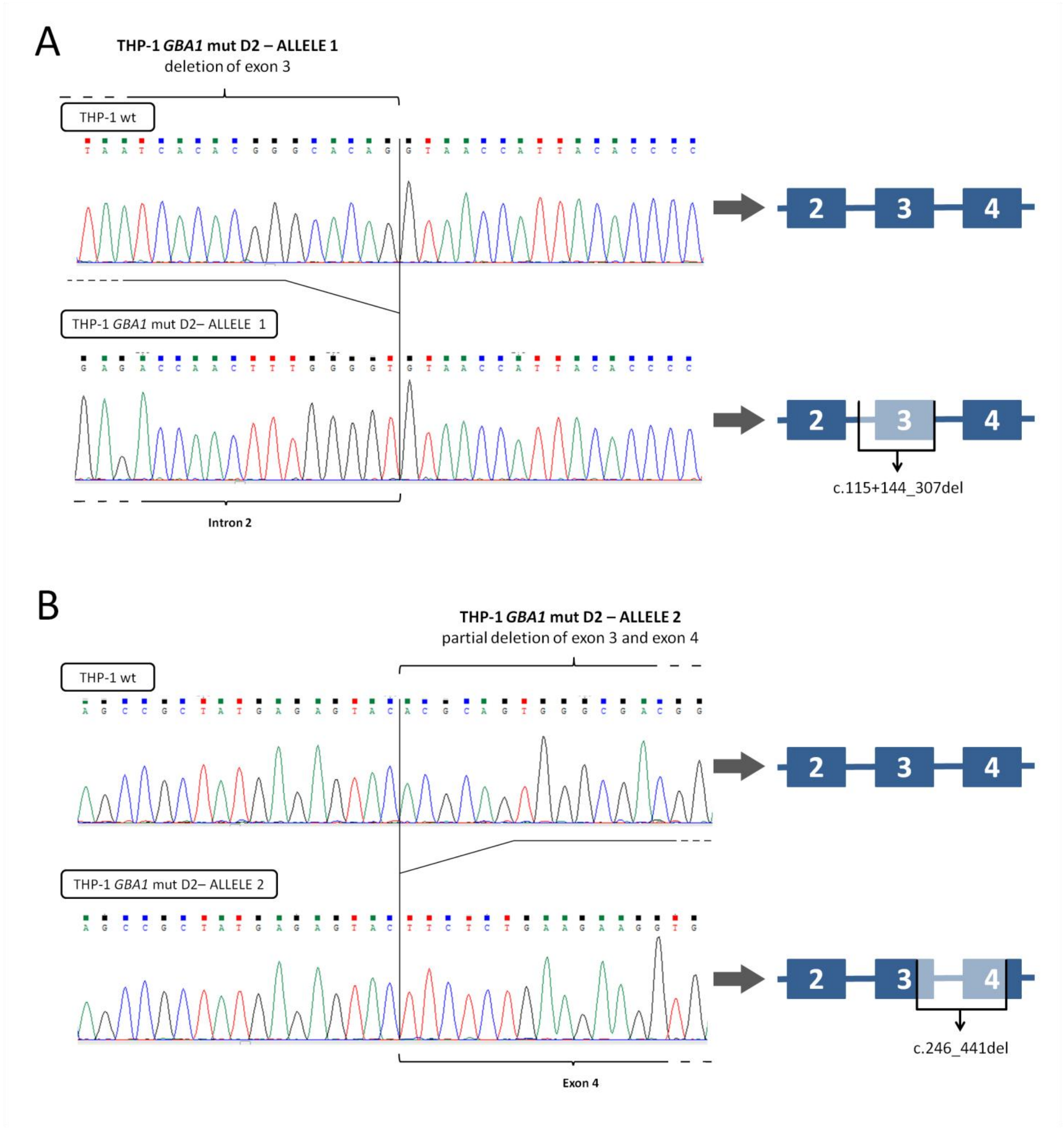

Figure S1. Sequence characterization of THP-1 *GBA1* mutant D2 | *GBA1* sequencing led to the identification of the two mutant alleles of THP-1 *GBA1* mutant D2 cells. (**A**) Allele 1: electropherogram shows the presence of a large in frame deletion causing the loss of the whole exon 3. (**B**) Allele 2: electropherogram shows the presence of a large deletion involving the final part of exon 3 and the first part of exon 4 (c.246_441del). [*Accession number of RNA sequence: NM_000157.3*].

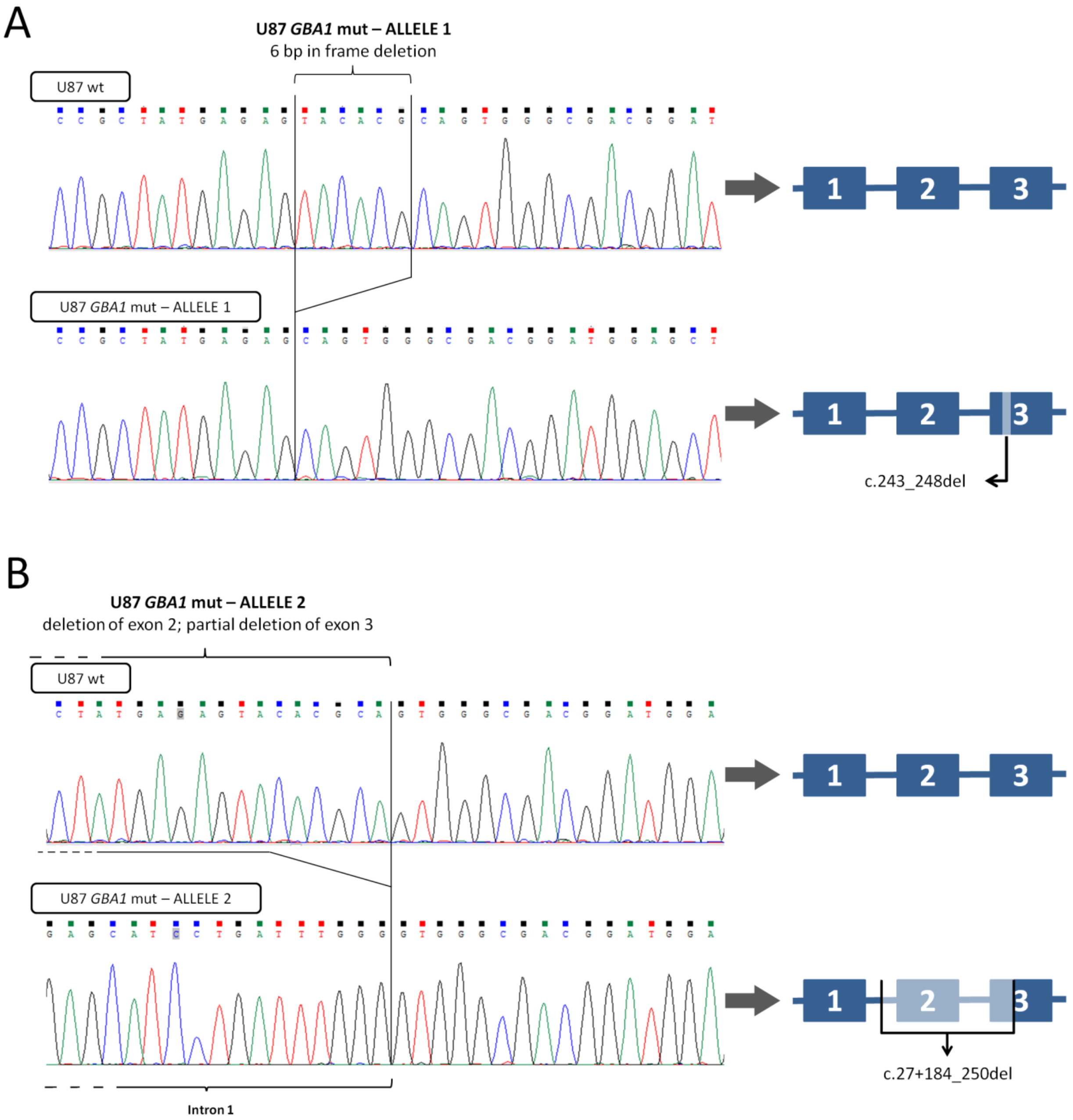

Figure S2. Sequence characterization of U87 *GBA1* mutant | *GBA1* sequencing led to the identification of the two mutant alleles of U87 *GBA1* mutant cells. Allele 1 (**A**) electropherogram shows a 6 bp in frame deletion within exon 3 (c.243_248del). Allele 2 (**B**) presents a large deletion involving the whole exon 2 and the first part of exon 3 (c.27+184_250del). [*Accession number of RNA sequence: NM_000157.3*].

Table S1: off-target analysis using the modified version of Zhang et al. [33] algorithm led to the identification of five exonic and nine intronic regions.

Location	Gene
Exonic	
Chr 3	*PTPN23*
Chr 12	*FMNL3/PRPF40B*
Chr 11	*DAK/DDB1*
Chr 1	*GBAP1*
Chr 19	*BCAT2*
Intronic	
Chr 15	*C15orf26*
Chr 14	*HSP90AA1*
Chr 7	*HEATR2*
Chr 8	*KCNK9*
Chr 11	*RP11-64I17.1*
Chr 5	*SLC6A3*
Chr 16	*CES1P1*
Chr 1	*RP11-139I14.2*
Chr 3	*SLCO2A1*

Table S2: Primers sequences

Primer	Sequence
1F	5'-CCTAAAGTTGTCACCCATAC-3'
5F	5'-AGCAGACCTACCCTACAGTTT-3'
3F	5'-GCAAGGCAGGTCTCAAACTC-3'
3R	5'-CCCTCCAAATCCCTTCACTT-3'
BiP F	5'-GGTACTGCTTGATGTATGTC-3'
BiP R	5'-GTCTTTCACCTTCATAGACC-3'
Chop F	5'-GACTTAAGTCTAAGGCACTG-3'
Chop R	5'-GATACACTTCCTTCTTGAACAC-3'
offtE-PTPN23 F	5'-CACGCCTTACACCTACCCTG-3'
offtE-PTPN23 R	5'-GTAGAGCTGGGTGTGTAGGG-3'
offtE-FMNL3/PRPF40B F	5'-GAGGGATGGAAGAGGGTGAG-3'
offtE-FMNL3/PRPF40B R	5'-TGCCCTGACACCTCTGATTC-3'
offtE-DAK/DDB1 F	5'-AATCCTGTGCTACCTGGGTC-3'
offtE-DAK/DDB1	5'-AGTGATGAAGTGGGTGCTGT-3'
offtE-BCAT2 F	5'-CACAGATGGGAAGGCATTGG-3'
offtE-BCAT2 R	5'-ATGTGGCTGAAAGGACCTGA-3'

offtI-HSP90AA1 F	5'-TGGGGTTTGGAGTGACTGAA-3'
offtI-HSP90AA1 R	5'-TTCTTTTGTCCTGTGGCTGC-3'
offtI-KCNK9 F	5'-GGTGATAACGTCCCTCCTCT-3'
offtI-KCNK9 R	5'-TCATCATCCTGCCCTGTGAG-3'
offtI-RP11-64I17.1 F	5'-GAGCTGCTCGTTAACATGGG-3'
offtI-RP11-64I17.1 R	5'-TGACCGCTAAGTACTGCCAT-3'
offtI-SLC6A3 F	5'-TACAGTTGTCTTGGCCCCAA-3'
offtI-SLC6A3 R	5'-TGTTTTCTGAGGGGATGGCT-3'
offtI-RP11-139I14.2 F	5'-TAGAAGCTGCTCCTCTGACC-3'
offtI-RP11-139I14.2 R	5'-ACATTGGCCCGCATTGTTC-3'
offtI-C15orf26 F	5'-CTAGGCTCCTGTTCTCCCAG-3'
offtI-C15orf26 R	5'-GGCTCAAGTATACCCAGGCT-3'
offtI-HEATR2 F	5'-GGCTGAAACCTGGAACCATG-3'
offtI-HEATR2 R	5'-TTTCCCGAAAAGCCACCATG-3'
offtI-CES1P1.A	5'-GGCAGGCAGAAGTCTTCCTA-3'
offtI-CES1P1.A R	5'-GACAAGAGGTGGACAGAGGT-3'
offtI-SLCO2A1 II F	5'-TCTCATCGTCTTGTGCTGGT-3'
offtI-SLCO2A1 II R	5'-CATGGATGACACGAAGCTGG -3'
